# Which is the most effective rescue treatment after the failure of mechanical thrombectomy for acute basilar artery occlusion?

**DOI:** 10.3389/fneur.2022.992396

**Published:** 2022-10-24

**Authors:** Jun Luo, Deping Wu, Zhimin Li, Dongjing Xie, Jiacheng Huang, Jiaxing Song, Weidong Luo, Shuai Liu, Fengli Li, Wenjie Zi, Qiaojuan Huang, Jiefeng Luo, Deyan Kong

**Affiliations:** ^1^Department of Neurology, Sichuan Mianyang 404 Hospital, Mianyang, China; ^2^Department of Neurology, Xinqiao Hospital, The Second Affiliated Hospital, Army Medical University (Third Military Medical University), Chongqing, China; ^3^Department of Neurology, Affiliated Hospital of North Sichuan Medical College, Nanchong, China; ^4^Department of Cardiology, The Second Affiliated Hospital of Guangxi Medical University, Nanning, China; ^5^Department of Neurology, The Second Affiliated Hospital of Guangxi Medical University, Nanning, China

**Keywords:** basilar artery occlusion, mechanical thrombectomy, rescue therapy, stroke, recanalization

## Abstract

**Objective:**

The aim of this study was to evaluate the effectiveness and safety of rescue therapy, a therapy in which rescue devices such as balloon angioplasty, Apollo stent, Wingspan stent, Solitaire stent, or other self-expanding stents are used after the failure of mechanical thrombectomy (MT) and to determine the most effective rescue measure for acute basilar artery occlusion (BAO) after the failure of MT.

**Methods:**

For this study, we recruited patients from the BASILAR registry. All participants were divided into three groups: the recanalized with rescue therapy group, the recanalized without rescue therapy group, and the non-recanalized group. Clinical outcomes at 90 days and 1 year were compared. The association of rescue measures with favorable outcomes (modified Rankin Scale [mRS] score of 0–3) in patients achieving successful recanalization *via* rescue therapy was estimated using multivariate logistic regression analyses.

**Results:**

Among the participants, recanalization failure was found in 112 patients and successful recanalization in 473 patients, with 218 patients receiving rescue therapy and 255 patients without rescue therapy. Of these, 111 (43.5%) patients in the recanalized without rescue therapy group, 65 (29.8%) patients in the recanalized with rescue therapy group, and nine (8.0%) patients in the non-recanalized group achieved favorable outcomes at 90 days. Both the recanalization with rescue therapy and the recanalization without rescue therapy groups were associated with favorable outcomes at 90 days and 1 year compared with the non-recanalized group. Moreover, in patients receiving rescue therapy, Wingspan stents, Apollo stents, and balloon angioplasty were associated with higher rates of favorable outcomes at 90 days and 1 year than Solitaire stents.

**Conclusion:**

Whether rescue therapy is administered or not, recanalization leads to favorable outcomes in patients with acute BAO. For acute BAO after MT failure, balloon angioplasty, Wingspan stenting, and Apollo stenting could be considered effective and safe rescue options but not Solitaire stenting.

## Introduction

Acute basilar artery occlusion (BAO) is one of the fatal diseases, with a mortality of 90% and extremely high risk of disability if left untreated. Endovascular treatment (EVT) is an important treatment for acute BAO in clinical practice. Recently, some prospective or retrospective cohort studies suggested that EVT could improve the prognosis in patients with acute BAO (1–3).

For recanalization, both stent retrievers and contact aspiration are highly effective in removing emboli; however, 20–40% of patients with BAO still require rescue treatment to sustain recanalization (4, 5). In pursuit of successful reperfusion, multiple stent retriever passes or prolonged contact aspiration is often practiced, which is associated with worse outcomes and longer procedure duration as rescue treatment may lead to perforator occlusion and reperfusion delay (6, 7). Hence, it is essential to achieve successful reperfusion with fewer numbers of stent retriever passes. Whether it is safe to use rescue treatment after recanalization failure is unclear. Various devices are available for rescue treatment, including balloon angioplasty, Apollo stent, Wingspan stent, Solitaire stent, and other off-label self-expanding stents (Neuroform EZ and Enterprise stent). However, the effect of these devices on the prognosis of patients with acute BAO remains unascertained.

Using the data from the Endovascular Treatment for Acute Basilar Artery Occlusion (BASILAR) study, we aimed to evaluate the impact of rescue therapy on short- and long-term outcomes of EVT in patients with acute BAO. We also aimed to determine the most effective rescue treatment for acute BAO after the failure of mechanical thrombectomy (MT).

## Methods

### Study design and participants

The BASILAR study was a nationwide prospective registry conducted in 47 comprehensive stroke centers in China between January 2014 and May 2019. The study protocol was approved by the ethics committee of the Xinqiao Hospital, Army Medical University, Chongqing, China as well as that of each subcenter. The BASILAR study was registered with the Chinese Clinical Trial Registry (http://www.chictr.org.cn; ChiCTR1800014759).

Patients were eligible for inclusion if they were 18 years or older with acute ischemic stroke caused by BAO within 24 h of estimated occlusion time, confirmed by computed tomographic angiography, magnetic resonance angiography, or digital subtraction angiography. Patients with cerebral hemorrhage, a premorbid modified Rankin Scale (mRS) score greater than 2, current pregnancy or lactation, or a serious, advanced, or terminal illness were excluded. Details of the study design have been published previously (1). We obtained written informed consent from patients or their legally authorized representatives according to the Declaration of Helsinki.

### Treatments and data collection

All eligible patients received EVT in combination with SMT. Treatment modalities of EVT, including mechanical thrombectomy with stent retrievers and/or thrombo-aspiration, balloon angioplasty, stenting, intra-arterial thrombolysis, or a combination of these approaches, were chosen at the discretion of neurointerventionalists. Rescue treatment can be defined as the use of rescue devices, such as balloon angioplasty, Apollo stent, Wingspan stent, Solitaire stent, and other self-expanding stents after the failure of MT. Based on recanalization with or without rescue therapy, the participants were divided into three groups: the non-recanalized group, the recanalized without a rescue therapy group, and the recanalized with a rescue therapy group.

The demographic characteristics, stroke risk factors, the premorbid modified Rankin Scale (mRS) score, the baseline National Institutes of Health Stroke Scale (NIHSS) score, and the baseline posterior circulation–Acute Stroke Program Early CT Score (pc-ASPECTS) were graded, as described earlier (8). The posterior circulation collateral score (PC-CS) represents the collateral circulation status based on the presence of potential collateral pathways on computed tomography angiography (9), the trial of Org 10172 in Acute Stroke Treatment (TOAST) classification, the location of the occlusion, intravenous thrombolysis, important time metrics, and the thrombolysis in cerebral infarction (TICI) score. A TICI score ≥2b was defined as successful recanalization (10).

### Outcome measures

The clinical outcomes we measured included short- and long-term outcomes. The primary outcome was a favorable functional outcome defined as an mRS score of 0–3 at 90 days. Secondary outcomes were functional independence (defined as an mRS score of 0–2) at 90 days, favorable outcomes at 1 year, mortality within 90 days or 1 year, re-occlusion within 24 h, and symptomatic intracerebral hemorrhage (sICH) within 48 h based on the Heidelberg Bleeding Classification.

### Statistical analysis

Categorical and binary variables were compared using χ^2^ tests (or Fisher exact tests), while continuous variables were compared using Student's *t*-test (mean comparison) and the Mann–Whitney *U* test for normal distribution variables. The Bonferroni test was used for multiple comparisons.

For baseline characteristics and outcomes, normally distributed continuous variables were presented as means and standard deviations, non-normally distributed continuous variables and ordinal variables were indicated as medians and interquartile ranges (IQRs), and categorical variables were indicated as absolute numbers and percentages. The effects of recanalization or rescue therapy on clinical outcomes were assessed using multivariable logistic regression, adjusting for age, history of diabetes, the baseline NIHSS score, baseline pc-ASPECTS, and occlusion site.

The second part only included patients achieving recanalization after rescue therapy. The clinical outcomes of different rescue measures were compared using the χ^2^ test or the Fisher exact test, with Bonferroni correction. Using the means of other variables, favorable outcomes for different rescue measures were predicted.

We plotted the probabilities of favorable functional outcomes, and then we presented adjusted odds ratios with 95% confidence intervals. Probabilities of predicted outcomes were presented as three-dimensional distribution surface diagrams generated by SigmaPlot 14 and assessed using the R^2^ correlation metric.

We performed statistical analyses using SPSS version 26.0 (IBM Corp., Armonk, NY, USA) and STATA version 16.0 (StataCorp LLC, TX, USA). The key variables in this study had low missingness and were analyzed with complete cases, but missing outcomes (sICH, 11 [1.9%]; mRS 0–2, mRS 0–3, and mortality at 1 year, 28 [4.8%]) were imputed using multiple imputations in the multivariate regression models. In the two-tailed test, a *P*-value < 0.05 was considered statistically significant.

## Results

### Patient characteristics

In the BASILAR registry involving 829 patients, we included 585 patients treated with EVT and with available mTICI scores. Among them, recanalization failure occurred in 112 patients and successful recanalization in 473 patients, with 218 patients receiving rescue therapy and 255 patients not receiving rescue therapy. The median (IQR) age and baseline NIHSS score of the entire cohort were 64 (56–74) years and 27 (7–33) years, respectively. A total of 147 patients (25.1%) were women and 438 patients (74.9%) were men.

A comparison of baseline characteristics of patients with endovascular thrombectomy in the recanalized with or without rescue therapy and non-recanalized groups is provided in Table 1. The patients in the recanalized without rescue therapy group had higher baseline pc-ASPECTS, more occlusion of the distal basilar artery (BA), less occlusion of the middle BA, higher frequency of atrial fibrillation, and shorter puncture-to-recanalization time. The patients in the non-recanalized group had higher baseline glucose.

**Table 1:** Baseline characteristics of the patients treated with endovascular thrombectomy in the recanalized with or without rescue therapy and the non-recanalized groups.

	**Recanalized without rescue therapy group** ** (*n =* 255)**	**Recanalized with rescue therapy group** ** (*n =* 218)**	**Non-recanalized group** ** (*n =* 112)**	***P*-value**
Age, y, median (IQR)	64 (56–74)	64 (57–72)	63 (53–73)	0.174
Women, n/total *n* (%)	74/255 (29.0)	42/218 (19.3)	31/112 (27.7)	0.040
Baseline NIHSS, median (IQR)	27 (17–33)	25 (15–32)	29 (19–35)	0.089
Baseline pc-ASPECTS, median (IQR)	8 (7–10)	8 (7–9)	7 (6–8)	0.000
GLU, median (IQR)	7.2 (5.9–9.1)	7.5 (6.1–9.7)	8.2 (6.6–10.9)	0.001
SBP, median (IQR)	148 (130–162)	150 (135–170)	150 (135–167)	0.123
Premorbid mRS, n/total *n* (%)				0.562
0	222 (87.1)	182 (83.5)	91 (81.3)	
1	22 (8.6)	25 (11.5)	16 (14.3)	
2	11 (4.3)	11 (5.0)	5 (4.5)	
TOAST, n/total *n* (%)				0.000
LAA	108 (42.4)	193 (88.5)	70 (62.5)	
CE	118 (46.3)	17 (7.8)	29 (25.9)	
Others	29 (11.4)	8 (3.7)	13 (11.6)	
History, n/total *n* (%)				
Ischemic stroke	44. (17.3)	57 (26.1)	24 (21.4)	0.063
Hypertension	162. (63.5)	160 (73.4)	82 (73.2)	0.039
Diabetes	51. (20.0)	55 (25.2)	28 (25.0)	0.339
Hyperlipidemia	81. (31.8)	73 (33.5)	42 (37.5)	0.563
Atrial fibrillation	93 (36.5)	14 (6.4)	22 (19.6)	0.000
Intravenous thrombolysis, n/total *n* (%)	55 (21.6)	43 (19.7)	28 (25.0)	0.544
Location of occlusion, n/total *n* (%)				0.000
Distal BA	138 (54.1%)	25 (11.5)	27 (24.1)	
Middle BA	57 (22.4)	76 (34.9)	45 (40.2)	
Proximal BA	28 (11.0)	54 (24.8)	21 (18.8)	
VA-V4	32 (12.5)	63 (28.9)	19 (17.0)	
Time metrics, min, median (IQR)				
Onset to puncture time	315 (218–457)	360 (233–541)	340 (215–496)	0.488
Puncture-to-recanalization time	79 (60–114)	128 (93–168)	120 (85–164)	0.000

IQR, interquartile range; NIHSS, National Institutes of Health Stroke Scale; pc-ASPECTS, posterior circulation–Acute Stroke Prognosis Early Computed Tomography Score; GLU, glucose; SBP, systolic blood pressure; mRS, modified Rankin Scale; TOAST, Trial of Org 10172 in Acute Stroke Treatment; LAA, large-artery atherosclerosis; CE, cardioembolism; BA, basilar artery; VA-V4, V4 of the vertebral artery.

### Clinical outcomes

**Figure 1:**
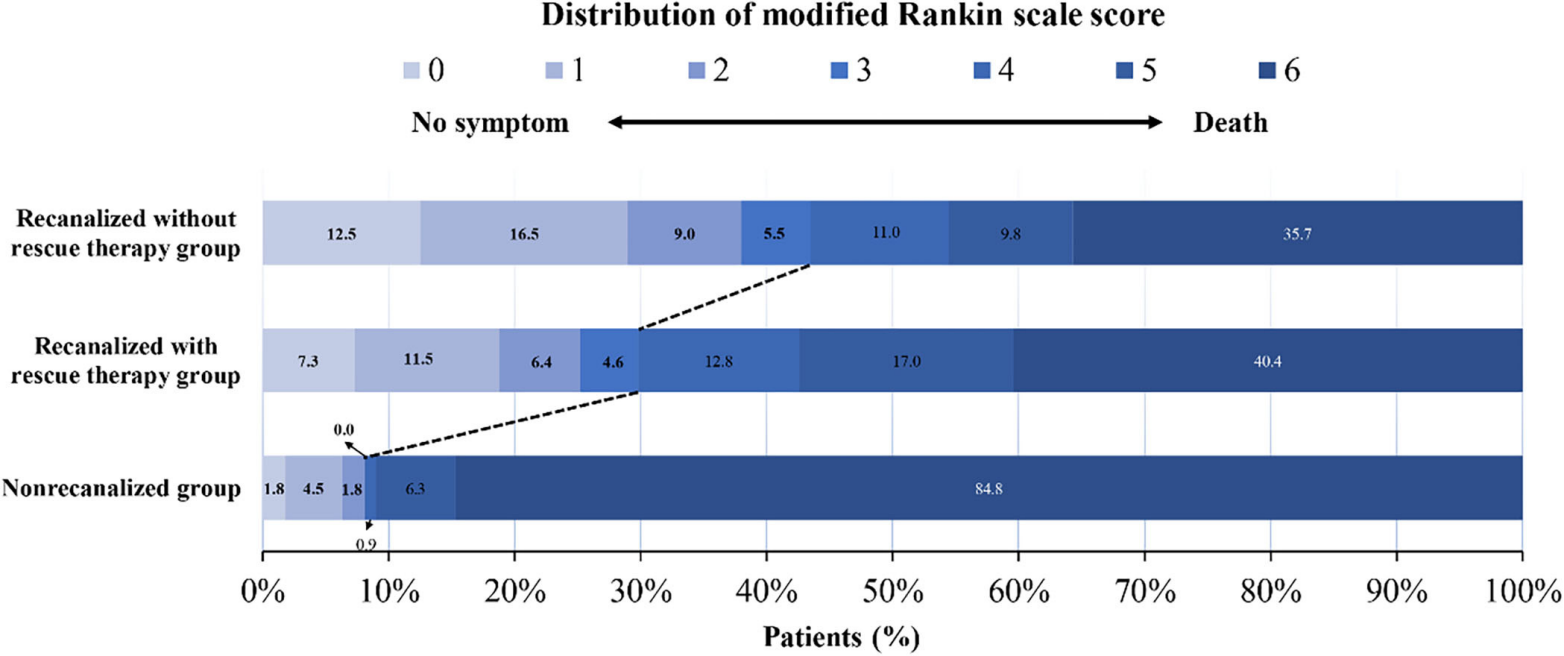
Distribution of scores on the modified Rankin Scale (mRS) at 90 days. The distribution of the mRS scores for favorable functional outcomes and mortality among patients in the non-recanalized group and in the recanalized with or without rescue therapy group. The numbers in the charts are percentages of patients who had each score, and the percentages may not sum to 100% owing to rounding.

mRS distributions at 90 days in the non-recanalized group, the recanalized without rescue therapy group, and the recanalized with rescue therapy group are shown in Figure 1. Among the patients treated with EVT, nine patients (8.0%) in the non-recanalized group, 111 patients (43.5%) in the recanalized without rescue therapy group, and 65 patients (29.8%) in the recanalized with rescue therapy group achieved favorable functional outcomes at 90 days (Table 2). Whether rescue therapy was administered or not, the patients with successful recanalization had higher rates of functional independence and favorable outcomes, lower mortality at 90 days and 1 year, and lower rates of sICH within 48 h than the patients in the non-recanalized group (all adjusted *P*-value < 0.05). Moreover, compared with the recanalized without rescue therapy group, the recanalized with rescue therapy group showed lower rates of functional independence and favorable outcomes at 90 days, and functional independence at 1 year (all adjusted *P*-value < 0.05).

**Table 2:** Comparisons of clinical outcomes among recanalized patients with or without rescue therapy and non-recanalized patients.

	**Overall**	**Non-recanalized group**	**Recanalized without rescue therapy group**	**Recanalized with rescue therapy group**	***P*-value^a^**	***P*-value^b^**	***P*-value^c^**	***P*-value^d^**
Clinical outcomes in the short term, n/total *n* (%)			
mRS 0–3 at 90 days	185/585 (31.6)	9/112 (8.0)	111/255 (43.5)	65/218 (29.8)	<0.001	<0.001	<0.001	0.003
mRS 0–2 at 90 days	161/585 (27.5)	9/112 (8.0)	97/255 (38.0)	55/218 (25.2)	<0.001	<0.001	<0.001	0.002
Mortality at 90 days	274/585 (46.8)	95/112 (84.8)	91/255 (35.7)	88/218 (40.4)	<0.001	<0.001	<0.001	0.342
SICH within 48 hours	38/574 (6.6)	15/106 (14.2)	14/255 (5.5)	9/213 (4.2)	0.002	**0.011**	**0.003**	0.678
Clinical outcomes in the long term, n/total *n* (%)
mRS 0–2 at 1 year	174/557 (31.2)	7/106 (6.6)	106/249 (42.6)	61/202 (30.2)	<0.001	<0.001	<0.001	0.003
mRS 0–3 at 1 year	201/557 (36.1)	9/106 (8.5)	115/249 (46.2)	77/202 (38.1)	<0.001	<0.001	<0.001	0.039
Mortality at 1 year	304/557 (54.6)	95/106 (89.6)	107/249 (43.0)	102/202 (50.5)	<0.001	<0.001	<0.001	0.134
Severe adverse events, n/total *n* (%)			
Pulmonary infection	439/585 (75.0)	83/112 (74.1)	187/255 (73.3)	169/218 (77.5)	0.558	0.979	0.579	0.344
Respiratory Failure	241/585 (41.2)	71/112 (63.4)	81/255 (31.8)	89/218 (40.8)	<0.001	<0.001	<0.001	0.051
Circulatory failure	140/585 (23.9)	47/112 (42.0)	46/255 (18.0)	47/218 (21.6)	<0.001	<0.001	<0.001	0.399
Gastrointestinal hemorrhage	104/585 (17.8)	26/112 (23.2)	34/255 (13.3)	44/218 (20.2)	0.037	0.028	0.620	0.061

Comparisons were made using the chi-square test with Bonferroni correction for multiple comparisons.

mRS, modified Rankin Scale; sICH, symptomatic intracranial hemorrhage.

a *P-*value for comparison among three groups.

b Adjusted *P*-value for the recanalized without rescue therapy group vs. non-recanalized group.

c Adjusted *P*-value for the recanalized with rescue therapy group vs. non-recanalized group.

d Adjusted *P*-value for the recanalized with rescue therapy group vs. recanalized without rescue therapy group.

In the multivariable analyses, compared with the non-recanalized group, the recanalized without rescue therapy group resulted in a 6.7-fold increased probability of 90-day favorable functional outcomes (adjusted OR, 7.7, 95% CI, 3.05 to 19.44, *P* < 0.001), and the recanalized with rescue therapy group resulted in a 5.85-fold increased probability of 90-day favorable functional outcomes (adjusted OR, 6.85, 95% CI, 2.68 to 17.50, *P* < 0.001). Compared with the non-recanalized group, the recanalized without rescue therapy and the recanalized with rescue therapy groups had other better short- and long-term outcomes. However, compared with the recanalized without rescue therapy group, the recanalized with rescue therapy group did not show worse short- and long-term outcomes (Table 3). The probability of favorable functional outcomes declined with the increase in the NIHSS score and puncture-to-recanalization time in the recanalized with and without rescue therapy groups, although there was no interaction between favorable outcomes, NIHSS, and puncture-to-recanalization time (Supplementary Figures 1A,B).

**Table 3:** Multivariable regression analyses of clinical outcomes at 90 days and 1 year.

	**Recanalized without rescue therapy group vs. Non-recanalized group**		**Recanalized with rescue therapy group vs. Non-recanalized group**		**Recanalized with rescue therapy group vs. Recanalized without rescue therapy group**	
	**Odds ratio (95% CI)**	***P*-value**	**Odds ratio (95% CI)**	***P*-value**	**Odds ratio (95% CI)**	***P*-value**
mRS 0–3 at 90 days	7.70 (3.05–19.44)	<0.001	6.85 (2.68–17.50)	<0.001	0.89 (0.52–1.52)	0.669
mRS 0–2 at 90 days	6.03 (2.36–15.39)	<0.001	5.28 (2.04–13.67)	0.001	0.88 (0.50–1.53)	0.642
Mortality at 90 days	0.10 (0.05–0.21)	<0.001	0.10 (0.05–0.21)	<0.001	1.01 (0.59–1.71)	0.980
SICH within 48 hours	0.37 (0.14–0.97)	0.043	0.28 (0.11–0.72)	0.008	0.74 (0.27–2.02)	0.558
mRS 0–3 at 1 year	8.75 (3.58–21.37)	<0.001	7.77 (3.18–18.99)	<0.001	0.89 (0.52–1.53)	0.888
mRS 0–2 at 1 year	10.02 (3.67–27.39)	<0.001	8.14 (2.96–22.42)	<0.001	0.81 (0.47–1.41)	0.462
Mortality at 1 year	0.10 (0.04–0.22)	<0.001	0.11 (0.05–0.25)	<0.001	1.13 (0.66–1.94)	0.663

Adjusted for sex, pc-ASPECTS, GLU, TOAST, hypertension, Atrial fibrillation, location of occlusion, and puncture-to-recanalization time.

CI, confidence interval; mRS, modified Rankin Scale; SICH, symptomatic intracranial hemorrhage; pc-ASPECTS, posterior circulation–Acute Stroke Prognosis Early Computed Tomography Score; GLU, glucose; TOAST, Trial of Org 10172 in Acute Stroke Treatment.

### Rescue measures and clinical outcomes

According to the rescue devices used, the patients treated with rescue therapy were divided into four subgroups: balloon angioplasty, Apollo stent, Solitaire stents, and other self-expanding stents. No difference was found among the characteristics of patients using different rescue devices (Supplementary Table 1).

**Figure 2:**
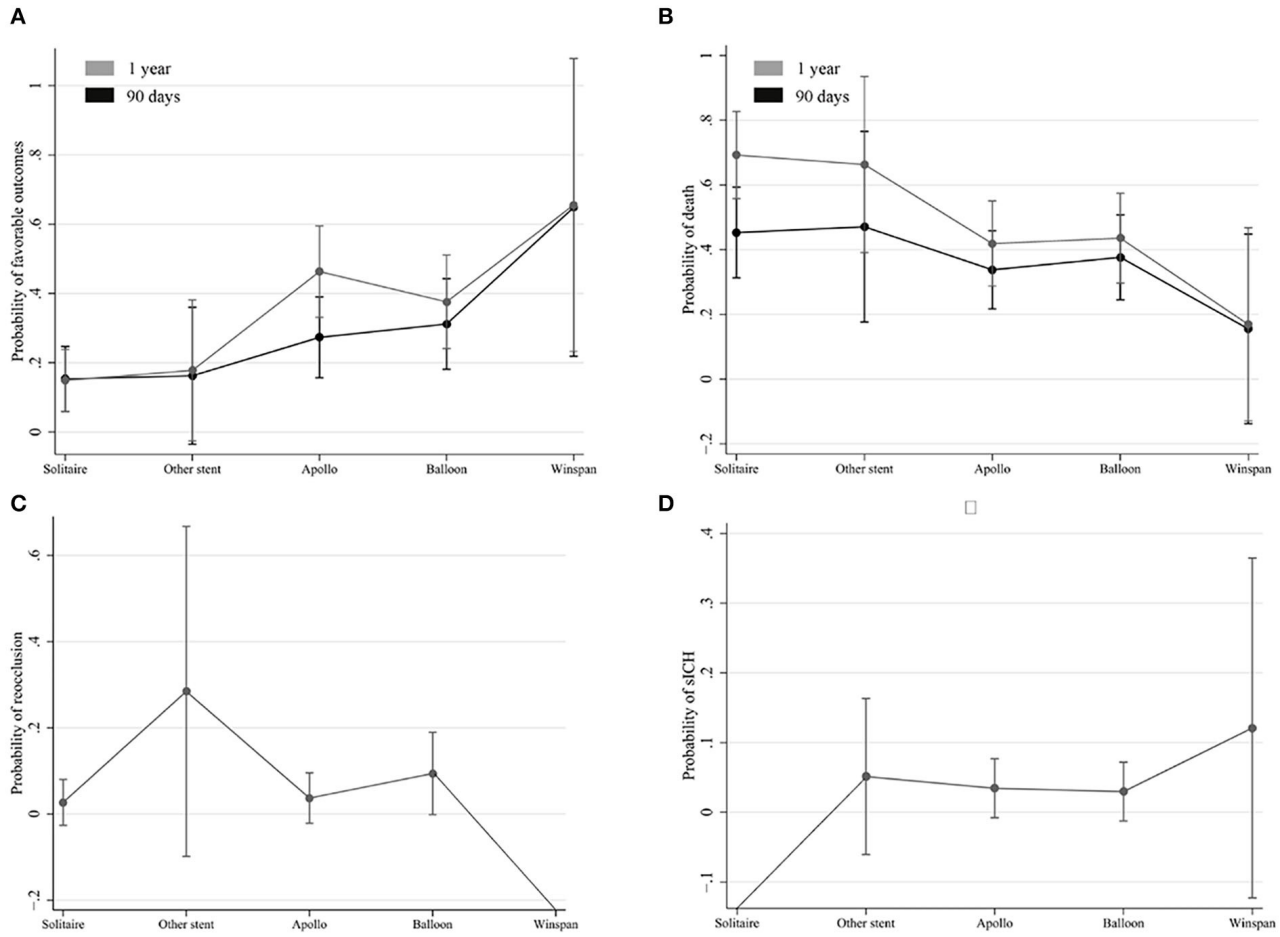
Estimated marginal effects of favorable outcome probabilities on different rescue measures. The estimated marginal effects of favorable outcome probabilities **(A)** and mortality **(B)** on different rescue measures in 90 days and 1 year. The estimated marginal effects of reocclusion probabilities **(C)** and sICH **(D)** on different rescue measures.

As shown in Table 4, the Solitaire stent had a significantly lower rate of favorable outcomes at 90 days than balloon angioplasty and other self-expanding stents. Also, the Solitaire stent had a significantly higher rate of mortality at 90 days than the Apollo stent and other self-expanding stents. Although the Solitaire stent had numerically less favorable outcomes and higher mortality rates at 1 year than other rescue devices, the differences were not statistically significant. The comparisons of re-occlusion within 24 h and sICH within 48 h among the four rescue devices were not significant (Table 4). Similar trends were observed in analyses of the occlusion of the middle BA and in analyses of the occlusion of the proximal BA or segment 4 of the vertebral artery, but trends were not significant due to small sample sizes (Supplementary Tables 2, 3). Different rescue measures have a direct effect on the probability of favorable functional outcomes. The estimated marginal effects of favorable outcome probability on the Wingspan and Apollo stents were higher than those of other rescue measures in 1 year, but not in 90 days. Furthermore, a lower risk of mortality and reocclusion was found in rescue therapy with Wingspan and Apollo stents (Figure 2).

**Table 4:** Comparison of clinical outcomes among patients rescued with different rescue measures.

	**Balloon**	**Apollo**	**Solitaire**	**Other self-expanding stent**	***P*-value^a^**	***P*-value^b^**	***P*-value^c^**	***P*-value^d^**
mRS 0–3 at 90 days	19/59 (32.2)	20/68 (29.4)	12/51 (23.5)	12/34 (35.3)	0.717	0.036	0.692	0.020
Mortality at 90 days	24/59 (40.7)	26/68 (38.2)	24/51 (47.1)	11/34 (32.4)	0.867	0.236	0.024	0.016
Reocclusion within 24h	2/34 (5.9)	2/36 (5.6)	3/28 (10.7)	2/20 (10.0)	0.937	0.252	0.795	0.230
SICH within 48h	3/57 (5.3)	4/67 (6.0)	0	2/32 (6.3)	0.690	0.227	0.107	0.255
mRS 0–3 at 1 year	22/55 (40.0)	30/62 (48.4)	9/45 (20.0)	14/34 (41.2)	0.719	1.000	1.000	1.000
Mortality at 1 year	25/55 (45.5)	28/62 (45.2)	32/45 (71.1)	13/34 (38.2)	0.726	0.383	0.125	0.104

Comparisons were made using the chi-square test with Bonferroni correction for multiple comparisons.

mRS, modified Rankin Scale; SICH, symptomatic intracranial hemorrhage.

*P*-value^a^, balloon angioplasty vs. Apollo stents.

*P*-value^b^, balloon angioplasty vs. solitaire stents.

*P*-value^c^, Apollo stents vs. solitaire stents.

*P*-value^d^, other self-expanding stents vs. solitaire stents.

## Discussion

This study demonstrated that recanalization with or without rescue therapy led to better clinical outcomes in patients with acute BAO. Recanalization with rescue therapy showed clinical outcomes similar to recanalization without rescue therapy. Based on these outcomes, it seemed reasonable and necessary to use rescue therapy for acute BAO after the failure of MT. Moreover, rescue therapy with balloon angioplasty, Wingspan stenting, or Apollo stenting might be effective and safe rescue options, but not with the Solitaire stent, for acute BAO after MT failure.

Our findings confirmed the safety of using rescue therapy after the failure of MT for acute BAO, which was considered clinically meaningful. To our knowledge, the frequency of recanalization was considered a practical and useful clinical marker in the delivery of EVT. As reported in previous studies, a strong correlation was found between successful recanalization and favorable outcomes at 3 months in non-recanalized patients with acute ischemic stroke (11, 12). Moreover, as reported in previous studies, early recanalization was confirmed as a strong predictor of good outcomes in patients undergoing either EVT or SMT after acute stroke with large-vessel occlusion (13, 14). However, the relationship between rescue therapy and good outcomes remains controversial. Lazzaro et al. demonstrated that rescue therapy was associated with poor outcomes due to a longer recanalization time and a lower percentage of successful recanalization in patients undergoing rescue therapy (7). Nevertheless, Jia et al. reported that mechanical thrombectomy in conjunction with a standard rescue therapy could achieve favorable outcomes in patients with intracranial large-artery occlusion (ILAO) with underlying intracranial atherosclerosis (ICAS) (15). Moreover, the data from observational studies indicated that rescue therapy with intracranial angioplasty and/or stenting was safe and efficacious in patients with emergent LAO with underlying ICAS (16). Our study demonstrated that patients with acute BAO could benefit from rescue therapy after the failure of MT, corroborating the clinical application of rescue therapy for acute BAO.

Our study indicated that rescuing with balloon angioplasty alone after the failure of MT achieved a good outcome at 90 days, which was consistent with a previous report (17). To our surprise, long-term outcome data demonstrated that, when the follow-up period was extended to 1 year, the clinical outcome of rescuing with balloon angioplasty alone was still not inferior to that of rescue stenting after the failure of MT in patients of acute BAO, corroborating the clinical use of balloon angioplasty alone as the rescue therapy for acute BAO after the failure of MT. As compared with rescue stenting, balloon angioplasty alone has several advantages. Balloon angioplasty alone could prevent vessel damage such as perforator occlusion and in-stent thrombosis and reduce the postoperative use of dual antiplatelet medication, which might help explain the benefits of rescuing with balloon angioplasty alone on short- and long-term outcomes in the patients with acute BAO. Our results suggest that permanent stenting might not be necessary if sufficient blood flow was successfully achieved and maintained with balloon angioplasty, in line with previous reports (18, 19).

In addition to balloon angioplasty, our subgroup analysis also suggested that Wingspan stenting or Apollo stenting seems to be an effective and safe option for acute BAO after the failure of MT, but the permanent placement of a Solitaire stent is not recommended for acute BAO after the failure of MT. Our results indicated that the short-term clinical outcomes of rescuing with Wingspan stent were superior to those of rescuing with Apollo stent, particularly in patients with occlusion of the middle BA, while the long-term outcomes of these two groups were comparable. It is well known that the BA, especially the middle segment of the BA, may be the most high-risk location for perforator stroke as it may involve a great number of perforators (20). With the Wingspan stent, a self-expanding, laser-cut, nitinol stent designed specifically for intracranial stenosis, it was easier to access and deliver to the target vessel with reduced barotrauma due to its flexibility and appropriate radial force, which theoretically decreases the risk of perforator occlusion and parent vessel dissection or rupture compared with balloon-expandable stents (21), which may help explain the main advantage of using Wingspan stents as rescue therapy in this trial. The Apollo stent is a balloon-expandable stent designed specifically for intracranial stenosis. The use of the balloon-expandable stent with a higher radial force than the Wingspan stent would more likely result in perioperative complications such as vasospasm, arterial dissection, or perforator occlusion (22), which can explain the worse outcome of rescuing with Apollo stents at 90 days. However, as these patients with perioperative complications recovered, it can be considered that using the rescue therapy with Apollo stents achieved comparable long-term clinical outcomes as compared with Wingspan stents in this trial, which was consistent with previous research (23). To save cost, the Solitaire stent was often selected as the rescue implant stent in clinical practices, particularly in developing countries. Nevertheless, according to our results, using the rescue therapy with permanent Solitaire stent implantation led to worse clinical outcomes than using the rescue therapy with balloon angioplasty, Wingspan stent, or Apollo stent. This result can be supported by several pieces of evidence. First, the Solitaire stent—designed as a stent-assisted coiling of aneurysms with a lower radial force and lacking adhesive force against the vessel wall when compared with Wingspan stents—was more likely to cause in-stent acute thrombosis. Second, as reported in a previous study (24), the Solitaire stent was rarely expanded fully. Therefore, after the detachment of the rescue Solitaire stent, on the one hand, the thrombus was easy to form near to or outside the strut, and, on the other hand, it was difficult to achieve sufficient blood flow, which are the reasons contributing to poor outcomes in patients. Due to these reasons, our study suggested that the Solitaire stent cannot be recommended as a rescue implant stent for acute BAO after MT failure.

There are several limitations to this study. First, the device selection was not randomized, and the rescue stent was selected based on the neurointerventionists' preference in each center. Second, the number of patients who underwent rescue therapy with Wingspan stents was small; therefore, further evaluation of a larger cohort will be necessary.

## Conclusion

The present study demonstrated that it is reasonable and necessary to administer rescue therapy after the failure of MT for acute BAO. This study also suggested that rescue angioplasty, Wingspan stenting, and Apollo stenting are effective and safe rescue options, but not the Solitaire stent, for acute BAO after MT failure. Future randomized clinical trials with a larger sample size of patients with BAO undergoing EVT are required to illuminate the effect of different rescue measures on the clinical prognosis of the patients.

## Data availability statement

The raw data supporting the conclusions of this article will be made available by the authors, without undue reservation.

## Ethics statement

The studies involving human participants were reviewed and approved by the Ethics Committee of the Xinqiao Hospital, Army Medical University. The patients/participants provided their written informed consent to participate in this study.

## Author contributions

JL, DW, and ZL interpreted the data and drafted the manuscript. JFL and DK contributed to the conception and design of the study. DX, JH, and JS performed the statistical analyses. WL and SL contributed to the acquisition, analysis, and interpretation of data. FL, WZ, and QH provided technical or material support and critically revised the manuscript. All authors contributed to the article and approved the submitted version.

## Funding

This research was supported by the National Natural Science Foundation of China (Grant No.: 81801361), the Health Commission of Sichuan Province (Grant No.: 18PJ337), and the Scientific Research Project of Guangxi Health Commission (Nos. Z-A20220669 and Z-A20220666). The sponsors were not involved in the study design, data collection, analysis and interpretation, writing, or decision to submit the article for publication.

## Conflict of interest

The authors declare that the research was conducted in the absence of any commercial or financial relationships that could be construed as a potential conflict of interest.

## Publisher's note

All claims expressed in this article are solely those of the authors and do not necessarily represent those of their affiliated organizations, or those of the publisher, the editors and the reviewers. Any product that may be evaluated in this article, or claim that may be made by its manufacturer, is not guaranteed or endorsed by the publisher.
