# Supplementary material for: Which is the most effective rescue treatment after the failure of mechanical thrombectomy for acute basilar artery occlusion?
Source: Front Neurol. 2022 Oct 24;13:992396. doi: 10.3389/fneur.2022.992396 (PMC9637633; doi:10.3389/fneur.2022.992396)

**Supplemental Material**

Table S1: Baseline characteristics of the patients treated with different rescue devices after failure of MT

|  | Balloon | Apollo | Solitaire | Other self-expanding stent | P value |
| --- | --- | --- | --- | --- | --- |
| Age, y, median (IQR) | 66 (58-73) | 65 (55-75) | 63 (57-72) | 63 (58-66) | 0.663 |
| Women, n/total n (%) | 11/59 (18.6) | 8/68 (11.8) | 13/51 (25.5) | 8/34 (18.9) | 0.242 |
| Baseline NIHSS, median (IQR) | 28 (15-34) | 25 (16-34) | 22 (15-30) | 24 (17-31) | 0.313 |
| Baseline pc-ASPECTS, median (IQR) | 8 (6-9) | 8 (7-9) | 8 (7-9) | 8.5 (7-9) | 0.490 |
| GLU, median (IQR) | 7.6 (6.5-9.9) | 7.8 (6.1-10.7) | 6.9 (5.8-8.3) | 7.2 (5.4-8.8) | 0.172 |
| SBP, median (IQR) | 150 (137-170) | 148 (128-163) | 159 (136-180) | 157 (138-175) | 0.161 |
| TOAST, n/total n (%) |  |  |  |  | 0.908 |
| LAA | 54/59 (91.5) | 60/68 (88.2) | 46/51 (90.2) | 33/34 (97.1) |  |
| CE | 3/59 (5.1) | 4/68 (5.9) | 3/51 (5.9) | 1/34 (2.9) |  |
| others | 2/59 (3.4) | 4/68 (5.9) | 2/51 (3.9) | 0 |  |
| History, n/total n (%) |  |  |  |  |  |
| Ischemic stroke | 18/59 (30.5) | 15/68 (22.1) | 16/51 (31.4) | 7/34 (20.6) | 0.492 |
| Hypertension | 48/59 (81.4) | 49/68 (72.1) | 34/51 (66.7) | 25/34 (73.5) | 0.366 |
| Diabetes | 19/59 (32.3) | 17/68 (25.0) | 8/51 (15.7) | 11/34 (32.4) | 0.191 |
| Hyperlipidemia | 17/59 (28.8) | 20/68 (29.4) | 24/51 (47.1) | 10/34 (29.4) | 0.135 |
| Intravenous thrombolysis, n/total n (%) | 12/59 (20.3) | 9/68 (13.2) | 4/51 (7.8) | 5/34 (14.7) | 0.305 |
| Location of occlusion, n/total n (%) |  |  |  |  | 0.726 |
| Distal BA | 7/59 (11.9) | 6/68 (8.8) | 3/51 (5.9) | 4/34 (11.8) |  |
| Middle BA | 21/59 (35.6) | 19/68 (27.9) | 23/51 (45.1) | 13/34 (38.2) |  |
| Proximal BA | 16/59 (27.1) | 18/68 (26.5) | 11/51 (21.6) | 9/34 (26.5) |  |
| VA-V4 | 15/59 (25.4) | 25/68 (36.8) | 14/51 (27.5) | 8/34 (23.5) |  |
| Time metrics, min, median (IQR) |  |  |  |  |  |
| Onset to puncture time | 331 (226-504) | 348 (223-624) | 384 (255-515) | 399 (262-602) | 0.631 |
| Puncture to recanalization time | 131 (99-175) | 122 (87-148) | 147 (97-184) | 107 (137-161) | 0.177 |

Abbreviations: IQR，interquartile range; NIHSS, National Institutes of Health Stroke Scale; pc-ASPECTS, posterior circulation–Acute Stroke Prognosis Early Computed Tomography Score; GLU, glucose; SBP, systolic blood pressure; mRS, modified Rankin Scale; TOAST, Trial of Org 10172 in Acute Stroke Treatment; LAA, large-artery atherosclerosis; CE, cardioembolism; BA, basilar artery; VA-V4, V4 of vertebral artery

**Table S2: The clinical outcomes of different remedial measures in occlusion of middle BA.**

|  | Balloon | Apollo | Solitaire | Other self-expanding stent | *P* value^a^ | *P* value^b^ | *P* value^c^ | *P* value^d^ |
| --- | --- | --- | --- | --- | --- | --- | --- | --- |
| mRS 0-3 at 90 days | 4/21 (19.0) | 5/19 (26.3) | 7/23 (30.4) | 6/13 (46.2) | > 0.05 | > 0.05 | > 0.05 | > 0.05 |
| 90-day mortality | 9/21 (42.9) | 4/19 (21.1) | 11/23 (47.8) | 3/13 (23.1) | > 0.05 | > 0.05 | > 0.05 | > 0.05 |
| reocclusion within 24h | 2/13 (15.4) | 1/12 (8.3) | 2/11 (18.2) | 1/9 (11.1) | > 0.05 | > 0.05 | > 0.05 | > 0.05 |
| SICH within 48h | 2/21 (9.5) | 0 | 0 | 0 | > 0.05 | > 0.05 | > 0.05 | > 0.05 |
| mRS 0-3 at 1year | 8/19 (42.1) | 9/15 (60.0) | 6/20 (30.0) | 7/13 (53.8) | > 0.05 | > 0.05 | > 0.05 | > 0.05 |
| 1-year mortality | 10/19 (52.6) | 5/15 (33.3) | 13/20 (65.0) | 4/13 (30.8) | > 0.05 | > 0.05 | > 0.05 | > 0.05 |

Comparisons were made using chi-square test with Bonferroni correction for multiple comparisons.

Abbreviations: mRS, modified Rankin Scale; SICH, symptomatic intracranial hemorrhage.

*P* value^a^, balloon angioplasty vs. Apollo stents;

*P* value^b^, balloon angioplasty vs. solitaire stents;

*P* value^c^, Apollo stents vs. solitaire stents;

*P* value^d^, other self-expanding stents vs. solitaire stents.

**Table S3: The clinical outcome of different remedial measures in occlusion of proximal BA and VA-V4.**

|  | Balloon | Apollo | Solitaire | Other self-expanding stent | *P* value^a^ | *P* value^b^ | *P* value^c^ | *P* value^d^ |
| --- | --- | --- | --- | --- | --- | --- | --- | --- |
| mRS 0-3 at 90 days | 11/31 (35.5) | 12/43 (27.9) | 5/25 (20.0) | 4/17 (23.5) | > 0.05 | > 0.05 | > 0.05 | > 0.05 |
| 90-day mortality | 12/31 (38.7) | 19/43 (44.2) | 12/25 (48.0) | 6/17 (35.3) | > 0.05 | > 0.05 | > 0.05 | > 0.05 |
| reocclusion within 24h | 0 | 1/21 (4.8) | 1/16 (6.3) | 1/9 (11.1) | > 0.05 | > 0.05 | > 0.05 | > 0.05 |
| SICH within 48h | 1/29 (3.4） | 4/42 (9.5) | 0 | 2/16 (12.5) | > 0.05 | > 0.05 | > 0.05 | > 0.05 |
| mRS 0-3 at 1year | 11/29 (37.9) | 18/41 (43.9) | 3/22 (13.6) | 6/17 (35.3) | > 0.05 | > 0.05 | > 0.05 | > 0.05 |
| 1-year mortality | 12/29 (41.4) | 20/41 (48.8) | 17/22 (77.3) | 6/17 (35.3) | > 0.05 | > 0.05 | > 0.05 | **< 0.05** |

Comparisons were made using chi-square test with Bonferroni correction for multiple comparisons.

Abbreviations: mRS, modified Rankin Scale; SICH, symptomatic intracranial hemorrhage.

*P* value^a^, balloon angioplasty vs. Apollo stents;

*P* value^b^, balloon angioplasty vs. solitaire stents;

*P* value^c^, Apollo stents vs. solitaire stents;

*P* value^d^, other self-expanding stents vs. solitaire stents.

**Figure S1: The effects of NIHSS and puncture to recanalization time on favorable outcomes in recanalized without rescue therapy group and recanalized with rescue therapy group.**


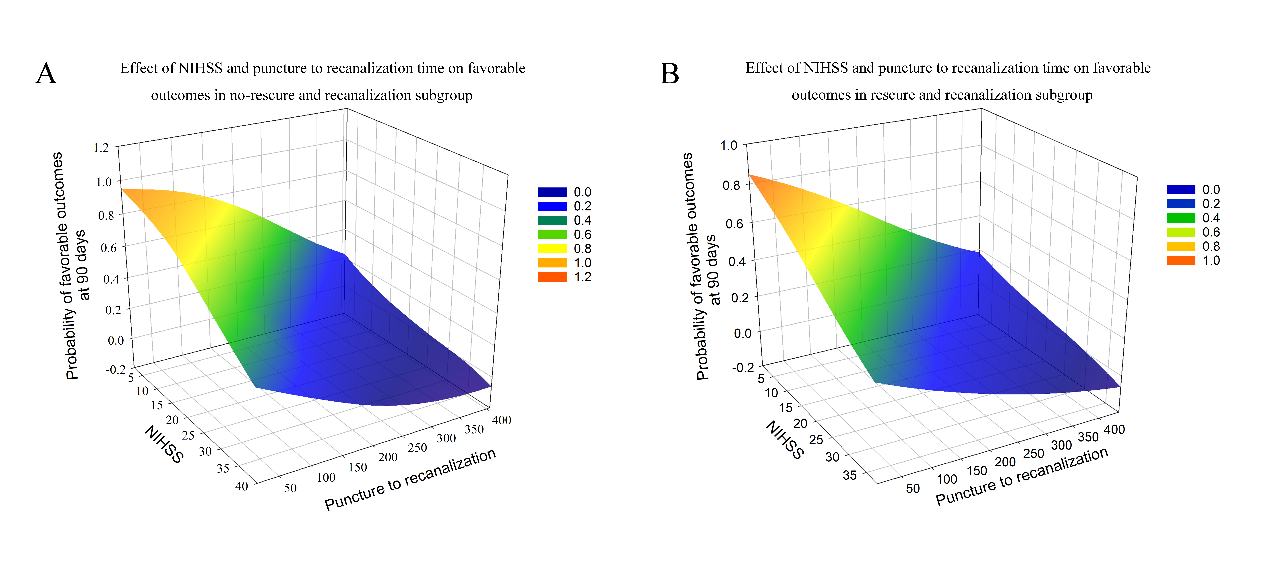

Supplement: Supplementary file 1 [file Data_Sheet_1.docx]
